# Corticotropin-Releasing Hormone (CRH) Promotes Macrophage Foam Cell Formation via Reduced Expression of ATP Binding Cassette Transporter-1 (ABCA1)

**DOI:** 10.1371/journal.pone.0130587

**Published:** 2015-06-25

**Authors:** Wonkyoung Cho, Jihee Lee Kang, Young Mi Park

**Affiliations:** 1 Department of Molecular Medicine, Ewha Womans University College of Medicine, Seoul, Republic of Korea; 2 Global Top 5 Research Group, Ewha Womans University, Seoul, Republic of Korea; 3 Department of Physiology, Tissue Injury Defense Research Center, Ewha Womans University College of Medicine, Seoul, Republic of Korea; University of Barcelona, SPAIN

## Abstract

Atherosclerosis, the major pathology of cardiovascular disease, is caused by multiple factors involving psychological stress. Corticotropin-releasing hormone (CRH), which is released by neurosecretory cells in the hypothalamus, peripheral nerve terminals and epithelial cells, regulates various stress-related responses. Our current study aimed to verify the role of CRH in macrophage foam cell formation, the initial critical stage of atherosclerosis. Our quantitative real-time reverse transcriptase PCR (qRT-PCR), semi-quantitative reverse transcriptase PCR, and Western blot results indicate that CRH down-regulates ATP-binding cassette transporter-1 (ABCA1) and liver X receptor (LXR)-α, a transcription factor for ABCA1, in murine peritoneal macrophages and human monocyte-derived macrophages. Oil-red O (ORO) staining and intracellular cholesterol measurement of macrophages treated with or without oxidized LDL (oxLDL) and with or without CRH (10 nM) in the presence of apolipoprotein A1 (apoA1) revealed that CRH treatment promotes macrophage foam cell formation. The boron-dipyrromethene (BODIPY)-conjugated cholesterol efflux assay showed that CRH treatment reduces macrophage cholesterol efflux. Western blot analysis showed that CRH-induced down-regulation of ABCA1 is dependent on phosphorylation of Akt (Ser473) induced by interaction between CRH and CRH receptor 1(CRHR1). We conclude that activation of this pathway by CRH accelerates macrophage foam cell formation and may promote stress-related atherosclerosis.

## Introduction

Cardiovascular disease remains the leading cause of death in many developed countries, for which atherosclerosis is the most important underlying pathology [1]. During the past decades, many studies have provided evidence that atherosclerosis is a multi-factorial disease involving interplay among many genetic and environmental factors [2]. Psychological factors have been regarded as an important indicator of atherosclerosis [3–5]. However, the mechanism by which psychological stress promotes atherosclerosis is not clearly defined.

Corticotropin-releasing hormone (CRH), a 41-amino-acid peptide known to be a stress hormone, links psychological stress to pathophysiologic responses [6]. CRH is released by neurosecretory cells of the paraventricular nucleus in the hypothalamus in response to various physical and psychological stressors, and blood CRH level has been shown to be higher in patients experiencing stress [7,8]. CRH stimulates secretion of the pituitary adrenocorticotropic hormone (ACTH) and subsequent release of adrenal steroids, which is called hypothalamo-pituitary adrenal (HPA) activation. CRH also functions in peripheral tissues, affecting the activities of immune cells such as monocytes, lymphocytes and neutrophils [9–13]. CRH activates resident immune cells in the inflammatory sites and performs pro-inflammatory functions in autocrine and paracrine manners [9–11].

Lipid-laden macrophages called foam cells play a crucial role in the initiation and progression of atherosclerosis [14]. Foam cells are generated by uncontrolled uptake of modified low-density lipoprotein (LDL), especially oxidized LDL (oxLDL), and/or impaired cholesterol efflux. Scavenger receptors, including CD36, mediate uptake of oxLDL and ATP-binding cassette (ABC) family transporters like ABCA1 and ABCG1, which mediate cholesterol efflux to apolipoprotein A1 (apoA1) and high-density lipoprotein (HDL) [15–17]. Therefore, lipid homeostasis in macrophages is maintained by the activities of these receptors and transporters. Alterations in the expression of these molecules may affect foam cell formation and the progression of atherosclerosis.

In the current study, we revealed that CRH promotes macrophage foam cell formation, which is the initial critical stage of atherosclerosis. Based on our experiments showing that CRH suppresses cholesterol efflux in macrophages via decreased expression of ABCA1, we suggest a mechanism by which CRH, the stress hormone, accelerates atherosclerosis and propose the CRH-provoking pathway as a new therapeutic target for the treatment of atherosclerosis.

## Materials and Methods

### Reagents

LDL was prepared from human plasma by density gradient ultracentrifugation [18]. Oxidatively modified LDL (oxLDL) was generated by dialysis of LDL with 5 μM CuSO4 in PBS for 6 hours at 37°C. Oxidation was terminated by dialysis against PBS containing EDTA (100 μM). LY294002 was purchased from Enzo (Enzo Life Sciences, NY, USA). NBI27914 hydrochloride was purchased from TOCRIS (Tocris Bioscience, Bristol, U.K.). CRH, oil red O (ORO), 1,1′-dioctadecyl-3,3,3′,3′-tetramethylindocarbocyanine perchlorate (DiI), human apolipoprotein A1 (apoA1), and human AB serum were purchased from Sigma (Sigma, St. Louis, MO, USA). The intracellular cholesterol assay kit was purchased from Cayman (Cayman Chemical Co., Ann Arbor, MI, USA). BODIPY-cholesterol was purchased from TopFluor (Avanti Polar Lipids, Inc., Alabaster, AL, USA). Antibodies against ABCA1 and EMR1(F4/80) were purchased from Abcam (Cambridge, MA, USA). Ficoll-paque was purchased from GE (GE Healthcare Bio-Sciences, Pittsburgh, PA, USA)

### Cell culture

Peritoneal macrophages of C57BL/6 male mice were collected by peritoneal lavage 4 days after intraperitoneal injection of 4% thioglycolate (1 ml). Mice were euthanized with CO_2_ before the collection of macrophages. About 30 mice were used to isolate macrophages as 1X10^7^ cells were collected from a mouse. Cells were cultured in RPMI containing 10% fetal bovine serum and 1% penicillin-streptomycin. Media was changed to serum-free RPMI for CRH or oxLDL treatment. Animal experiments for collecting macrophages were conducted in accordance with protocols approved by the Institutional Animal Care and Use Committee (IACUC) of Ewha Womans University College of Medicine (IACUC approval No. ESM-12-0198). Throughout the experiments, all efforts were made to minimize suffering.

Human monocytes were isolated from peripheral blood by Ficoll-Hypaque centrifugation [19] and were cultured in RPMI containing human AB serum (10%) for 7 days to allow for macrophage differentiation. The differentiation of the monocytes into macrophages was confirmed by flow cytometry with anti-EMR1(F4/80) antibody. Human peripheral blood was provided by the department of Laboratory Medicine (Blood Bank) in Ewha Womans University Medical Center under the Ewha Womans University Mokdong Hospital Internal Review Board (IRB)-approved protocol (12-20A-26). The sample was screened to confirm the absence of hepatitis B, hepatitis C and human immunodeficiency virus (HIV) infection. All experiments were performed with macrophages at 80% confluence; in 6-well plates with 1.0 X 10^6^ cells, in 12-well plates with 0.35 X 10^6^ cells or in 24-well plates with 0.15 X 10^6^ cells.

### Oil red O staining

For oil red O (ORO) staining, murine peritoneal macrophages were incubated with or without 50 μg/ml of oxLDL and with or without CRH (10 nM) for 18 hours. In some experiments, 10 μg/ml of apoA1 was added to the media. Then, macrophages were fixed in 4% paraformaldehyde for 20 minutes at room temperature. Cells were washed with PBS and then stained with 0.5% ORO solution for 30 min. After rinsing with distilled water, images were captured via microscopy. For measuring ORO-incorporated intracellular lipids, 500 μl of isopropanol was added to macrophages for 5 minutes to extract the ORO from the cells. Absorbance (optical density) at 510 nm was measured using spectrophotometry.

### Intracellular cholesterol measurement

Intracellular cholesterol measurement assay was performed following the instructions for the cholesterol fluorometric assay kit (Cayman Chemical Company). Briefly, macrophages were cultured in 6-well plates with or without CRH (10 nM), with or without oxLDL (50 μg/ml) and in with or without apoA1 (10 μg/ml). After 18 hours, we washed the cells twice with PBS and added assay buffer including 0.5% Triton X-100. The lysates of the macrophages were centrifuged at 13,000 rpm for 30 minutes at 4°C. Supernatants were used for the cholesterol measurement. The value of intracellular cholesterol was normalized by comparison to the protein concentration of the sample.

### BODIPY-cholesterol efflux assay

BODIPY-cholesterol efflux assay was performed as described in a previous study [20]. Briefly, macrophages cultured in 24-well plates (1.5 X 10^5^ cells/well) were labeled with BODIPY cholesterol labeling medium for 1 hour and washed with RPMI. Labeling medium was prepared through mixing unlabeled cholesterol (0.025mM), BODIPY- cholesterol (0.1 mM) and cyclodextrin (10 mM) in RPMI. Then, the cells were equilibrated with RPMI containing 2 μg/ml of acyl-coA:cholesterol acyltransferase (ACAT) inhibitor (Sandoz 58–035) for 18 hours and incubated with apoA1 (10 μg/ml) for 4 hours. The medium containing BODIPY-cholesterol excreted from the cells was analyzed via fluorometry (emission at 515 nm, excitation at 428 nm). The ABCA1-mediated BODIPY cholesterol efflux was measured by subtracting the fluorescence of the media of the cells without apoA1 from the fluorescence of the media of the cells with apoA1.

### 1,1′-Dioctadecyl-3,3,3′,3′-tetramethylindocarbocyanine perchlorate (DiI)-oxLDL uptake assay

LDL was labeled with DiI and oxidized as described previously [21]. Macrophages cultured on glass cover slips were treated with CRH for 18 hours and then with DiI-oxLDL (50 ug/ml) for 30–60 minutes. Cells were fixed in 4% paraformaldehyde in PBS and washed with PBS. DAPI staining was used to detect nuclei. DiI-oxLDL uptake of macrophages was evaluated using confocal fluorescence microscopy.

### RNA isolation, qRT-PCR and semi-quantitative reverse transcriptase-PCR

Total RNA was extracted from macrophages using Trizol reagent according to the manufacturer’s instructions (Invitrogen, Carlsbad, CA, USA). cDNA was synthesized using an iScript cDNA synthesis Kit (Bio-Rad, CA, USA). Quantitative real-time reverse transcriptase PCR (qRT-PCR) was performed with Power SYBR Green PCR Master Mix (Applied Biosystems, Foster City, CA, USA) and an ABI Real-Time PCR thermocycler. The primers for qRT-PCR using murine peritoneal macrophages are 5’-CCC AGA GCA AAA AGC GAC TC-3’ and 5’-GGT CAT CAT CAC TTT GGT CCT TG-3’ for ABCA1, 5’-CAA GAC CCT TTT GAA AGG GAT CTC-3’ and 5’-GCC AGA ATA TTC ATG AGT GTG GAC-3’ for ABCG1, 5’-GGC TGC TGT TTG CTG CG-3’ and 5’-GCT GCT TGA TGA GGG AGG G-3’ for SR-B1, 5’-GCT CTG CTC ATT GCC ATC AG-3’ and 5’-TGT TGC AGC CTC TCT ACT TGG A-3’ for LXR-α, 5’-GAT CGG AAC TGT GGG CTC AT-3’ and 5’-GGT TCC TTC TTC AAG GAC AAC TTC-3’ for CD36, 5’-AAA GAA GAA CAA GCG CAC GTG G-3’ and 5’-GAG CAC CAG GTG GAC CAG TTT G-3’ for SR-A, and 5’-TCC ATG ACA ACT TTG GCA TTG-3’ and 5’-TCA CGC CAC AGC TTT CCA-3’ for GAPDH. GAPDH was used as the internal control. Primers for human monocyte-derived macrophages are 5’-CCC AGA GCA AAA AGC GAC TC-3’ and 5’-GGT CAT CAT CAC TTT GGT CCT TG-3’ for ABCA1, 5’-CGG AGC CCA AGT CGG TGT G-3’ and 5’-TTT CAG ATG TCC ATT CAG CAG GTC-3’ for ABCG1, 5’-ACC GCA CCT TCC AGT TCC AG-3’ and 5’-ATC ACC GCC GCA CCC AAG-3’ for SR-B1, 5’-CTT GCT CAT TGC TAT CAG CAT CTT-3’ and 5’-ACA TAT GTG TGC TGC AGC CTC T-3’ for LXR-α, 5’-CTC TTT CCT GCA GCC CAA TG-3’ and 5’-GCT GCA GAA GAA TGT CAT TAA ATC TT-3’ for CD36, 5’-GCC AAC CTC ATG GAC ACA GA-3’ and 5’-GCT GCA GAA GAA TGT CAT TAA ATC TT-3’ for SR-A, 5’-CAA CGG ATT TGG TCG TAT TGG-3’ and 5’-GCA ACA ATA TCC ACT TTA CCA GAG TTA A-3’ for GAPDH. GAPDH was used as the internal control.

Semi-quantitative reverse transcriptase-PCR was performed with the following primers: 5’-AGT ACC CCA GCC TGG AAC TT-3’ and 5’-AGC TGT CCT TGG TCA GCT TC-3’ for ABCA1, 5’-GGA TAG GGT TGG AGT CAG CA-3’ and 5’-GG AGC GCC TGT TAC ACT GTT-3’ for LXR-α, 5’-ATG GTG AAG GTC GGT GTG-3’ and 5’-ACC AGT GGA TGC AGG GAT-3’ for GAPDH. After PCR, the results were normalized by comparison to GAPDH. Band intensities were quantified using ImageJ (http://rsbweb.nih.gov/ij). All PCR analyses were repeated at least 3 times.

### Immunoblot analyses

Murine peritoneal macrophages or human monocyte-derived macrophages were incubated in serum-free media with or without CRH (10–50 nM) or oxLDL (50 μg/ml) for 18 hours. Cells were then lysed in lysis buffer containing 20 mM Tris-HCl (pH 7.5), 150 mM NaCl, 1 mM EDTA, 1 mM EGTA, and 1% NP-40. A protease inhibitor cocktail (Roche, Mannheim, Germany) and phosphatase inhibitors (10 mM phenylmethylsulfonyl fluoride/PMSF, 1% sodium pyrophosphate, 10 mM sodium fluoride, and 2 mM sodium vanadate) were added. Twenty micrograms of protein were separated by SDS-polyacrylamide gel electrophoresis (PAGE), transferred to PVDF membranes (Millipore, Billerica, MA, USA), and analyzed by immunoblotting. Membranes were probed with ABCA1 antibody (Abcam). For immunoblotting to detect phospho-Akt (Ser473) (sc-33437, polyclonal, 1:1000) (Santa Cruz Biotechnology, Inc. Dallas, TX, USA), macrophages were incubated with 10 nM CRH for 5–60 minutes. β-actin antibody (C4) (sc-47778, monoclonal, 1:10000) (Santa Cruz Biotechnology, Inc.) was used for normalization. Band intensities were quantified using ImageJ (http://rsbweb.nih.gov/ij).

### Statistical analysis

Data are expressed as mean ± SEM. For statistical analysis, two-tailed Student’s t-test was used. *P* values less than 0.05 were considered significant. All experiments were repeated at least 3 times. In all panels, level of indicated protein or RNA normalized to β-actin or GAPDH was expressed as relative fold-increase compared to the control (untreated macrophages), which was arbitrarily set at 1 (100%). Analyses were performed using GraphPad Prism Software (Graphpad Software, La Jolla, CA, USA).

## Results

### CRH down-regulates ATP-binding cassette transporter-1(ABCA1) in murine and human macrophages

**Fig 1:**
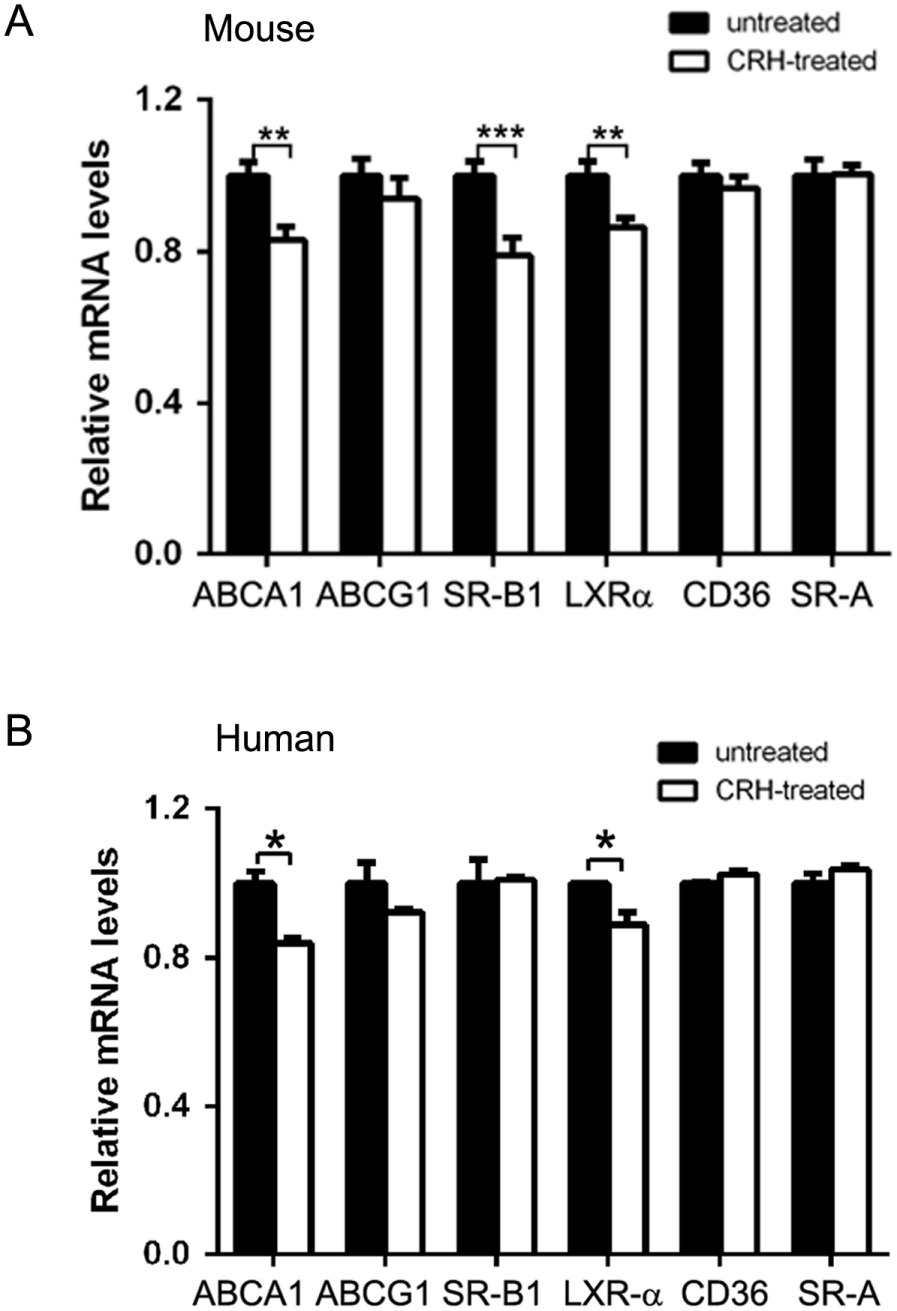
CRH down-regulates ABCA1 and LXR-α. (A) Quantitative real-time reverse transcriptase PCR (qRT-PCR) was performed with RNAs from murine peritoneal macrophages treated with or without CRH (10 nM) for 18 hours. GAPDH was used as the internal control. Relative mRNA levels of indicated molecules were compared between untreated (black bar) and CRH-treated (white bar) macrophages. (B) qRT-PCR was performed using RNAs from human peripheral blood monocyte-derived macrophages treated with or without CRH (20 nM) for 18 hours. GAPDH was used as the internal control. Relative mRNA levels of indicated molecules were compared between untreated (black bar) and CRH-treated (white bar) macrophages. Data are presented as mean ± SEM. * P < 0.*05*, ** P < 0.*01*, *** P < 0.*001*.

We performed assays to verify the effect of CRH on macrophage foam cell formation, the initial critical step in atherosclerosis (14). To systematically investigate the effect of CRH on macrophage lipid homeostasis, we performed quantitative real-time reverse transcriptase PCR (qRT-PCR) for molecules known to regulate macrophage lipid uptake and efflux. ABCA1, ABCG1 and SR-B1 are known to mediate cholesterol efflux of macrophages, while CD36 and Scavenger receptor-A (SRA) are involved in lipid uptake [15,17]. qRT-PCR results using murine peritoneal macrophages revealed that CRH treatment induced 20% decreases in ABCA1 and SR-B1 RNAs. Accordingly, liver X-receptor-α (LXR-α), a known transcription factor for ABCA1, also decreased by 18% in CRH-treated macrophages (Fig 1A). However, expression of the molecules mediating lipid uptake—CD36 and SRA—was not affected by CRH (Fig 1A).

qRT-PCR using human peripheral blood monocyte-derived macrophages showed 20% and 12% reductions in ABCA1 and LXR-α RNA levels, respectively. In contrast to the murine peritoneal macrophages, CRH did not decrease the RNA level of SR-B1 in human macrophages (Fig 1B).

### CRH induces a decrease in ABCA1 protein expression

**Fig 2:**
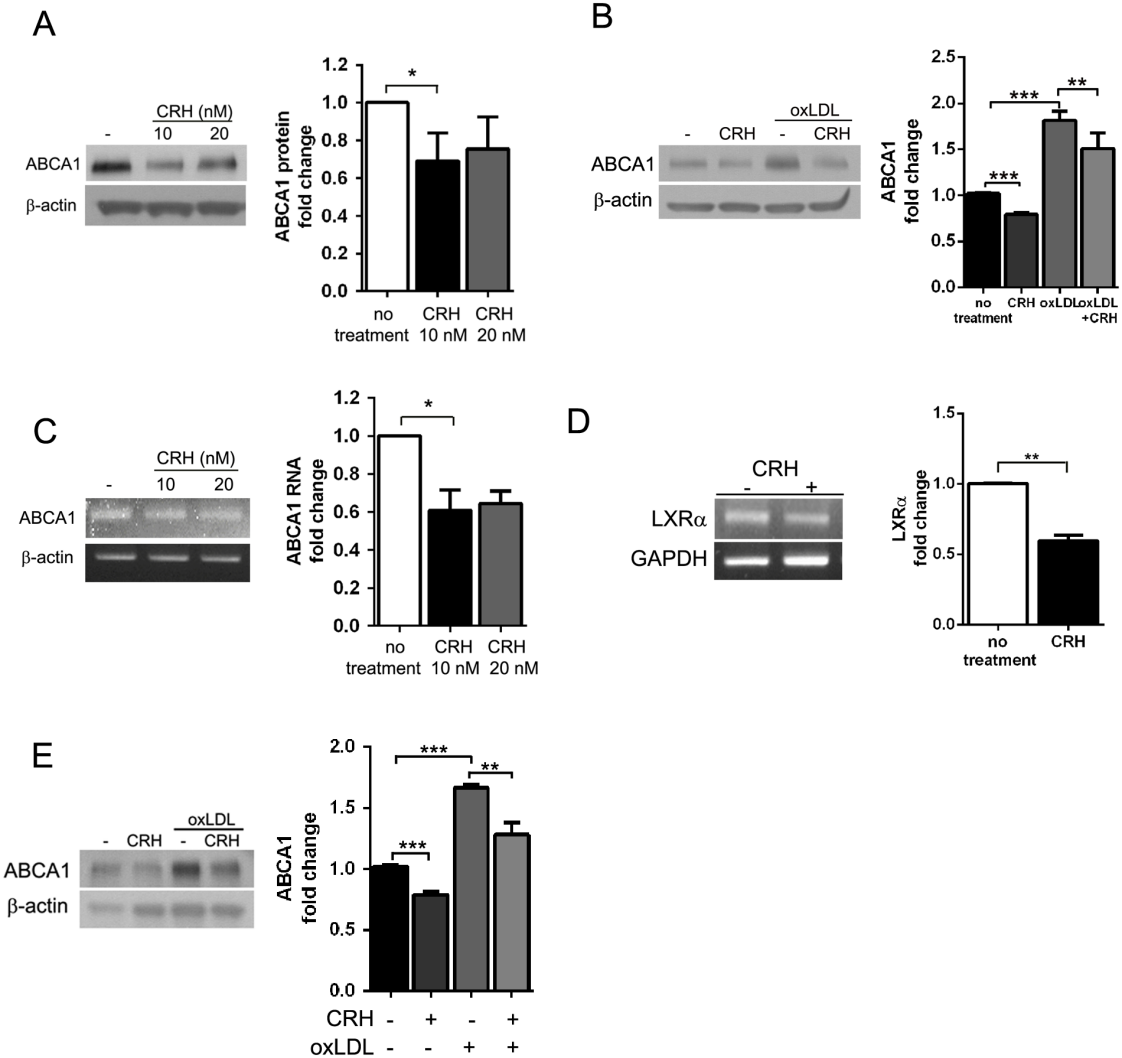
CRH induces a decrease in macrophage ABCA1 expression. (A) Western blot analysis for ABCA1 was performed using the cell lysates from murine peritoneal macrophages treated with or without CRH for 18 hours. (B) Western blot analysis for ABCA1 was performed using the cell lysates from murine peritoneal macrophages treated with or without CRH in the absence or presence of oxLDL (50 μg/ml). (C) Semi-quantitative reverse transcriptase-PCR for ABCA1. mRNA was extracted from macrophages treated as in (A). (D) Macrophages treated with or without CRH (10 nM) for 18 hours were lysed, and mRNA from these cells were utilized in semi-quantitative reverse transcriptase PCR for LXR-α. (E) Human monocyte-derived macrophages were treated with or without CRH (20 nM) in the absence or presence of oxLDL(50 μg/ml) for 18 hours. Western blot for ABCA1 was performed with the cell lysates. Quantitative data are presented as mean ± SEM (right graphs). * P < 0.*05*, ** P < 0.*01*, *** P < 0.*001*.

Based on the qRT-PCR results, we performed Western blot analysis for ABCA1 in order to determine the ABCA1 protein expression, revealing that murine peritoneal macrophages cultured with CRH for 18 hours demonstrated decreased ABCA1 expression (Fig 2A). OxLDL, which is abundant in atherosclerotic plaque, is known to increase ABCA1 expression in macrophages [22,23]. In our Western blot, CRH attenuated the ABCA1-increasing effect of oxLDL (Fig 2B). Semi-quantitative RT-PCR also confirmed a reduced ABCA1 RNA level in CRH-treated macrophages (Fig 2C).

We also confirmed the LXR-α down-regulating effect of CRH using semi-quantitative reverse transcriptase-PCR, showing a 40% decrease in LXR-α RNA in CRH-treated macrophages (Fig 2D), suggesting that CRH transcriptionally induces down-regulation of ABCA1.

Western blot analysis for ABCA1 in human monocyte-derived macrophages also presented a CRH-induced ABCA1 decrease. As in the experiment with murine macrophages, the Western blot analysis demonstrated that CRH attenuated the ABCA1 increasing effect of oxLDL (Fig 2E). Therefore, we concluded that CRH reduced ABCA1 expression in a transcriptional manner both in murine and human macrophages.

In accordance with our results from qRT-PCR, Western blot analysis for CD36 using murine peritoneal macrophages showed that CRH did not affect CD36 expression (S1 Fig).

### CRH promotes macrophage foam cell formation

**Fig 3:**
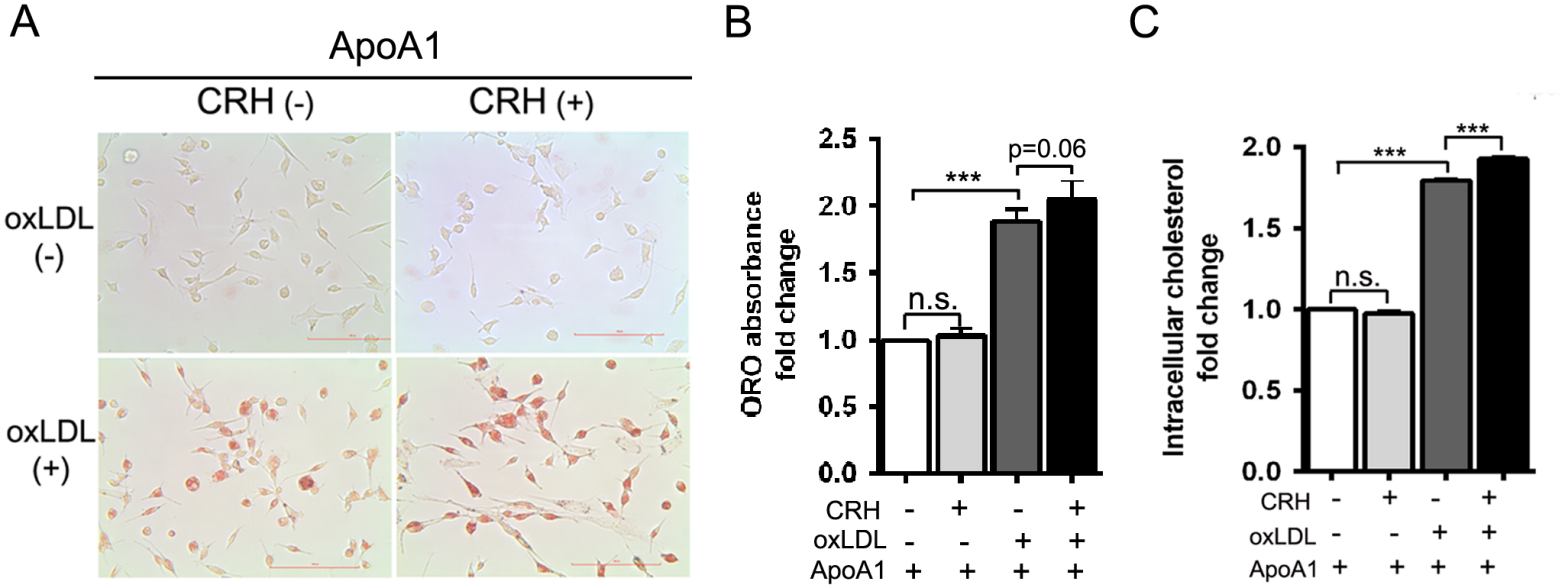
CRH promotes foam cell formation. (A) Murine peritoneal macrophages treated with or without CRH (10 nM) were incubated with or without oxLDL (50 μg/ml) for 18 hours in the presence of apoA1 (10 μg/ml). Oil-red-O (ORO) staining was performed. Representative pictures are provided (scale bar: 100 μm). (B) Measurement of ORO absorbance in the macrophage extracts. Fold-changes were plotted from triplicate experiments (C) Intracellular cholesterol measurement. After an 18-hour incubation with or without CRH (10 nM) and oxLDL (50 μg/ml), macrophages were lysed, and intracellular cholesterol was measured. All results are presented as mean ± SEM. The results were derived from triplicate experiments. *** P < 0.*001*, *n*.*s*.; *no statistical significance*.

Based on the data showing the CRH-induced down-regulation of ABCA1, we tested the effect of CRH on macrophage foam cell formation. Murine peritoneal macrophages treated with or without CRH (10 nM) were incubated with oxLDL (50 μg/ml) for 18 hours in the presence of apoA1 (10 μg/ml) and then stained with ORO (Fig 3A). Quantitation of the ORO incorporated into the intracellular lipids was performed by measuring the ORO in cellular extracts. The optical density of the ORO in the macrophage extract was increased by 1.8-fold after oxLDL treatment. CRH treatment induced an additional 10% increase in the optical density of the extract of oxLDL-treated macrophages (p = 0.06) (Fig 3B). The cholesterol assay measuring the amount of intracellular cholesterol in murine peritoneal macrophages treated with or without CRH and oxLDL revealed that oxLDL treatment induced a 1.8-fold increase of intracellular cholesterol. Furthermore, CRH addition induced an additional 6% increase of intracellular cholesterol in oxLDL-treated macrophages (p<0.001) (Fig 3C).

### Macrophage cholesterol efflux was reduced by CRH

**Fig 4:**
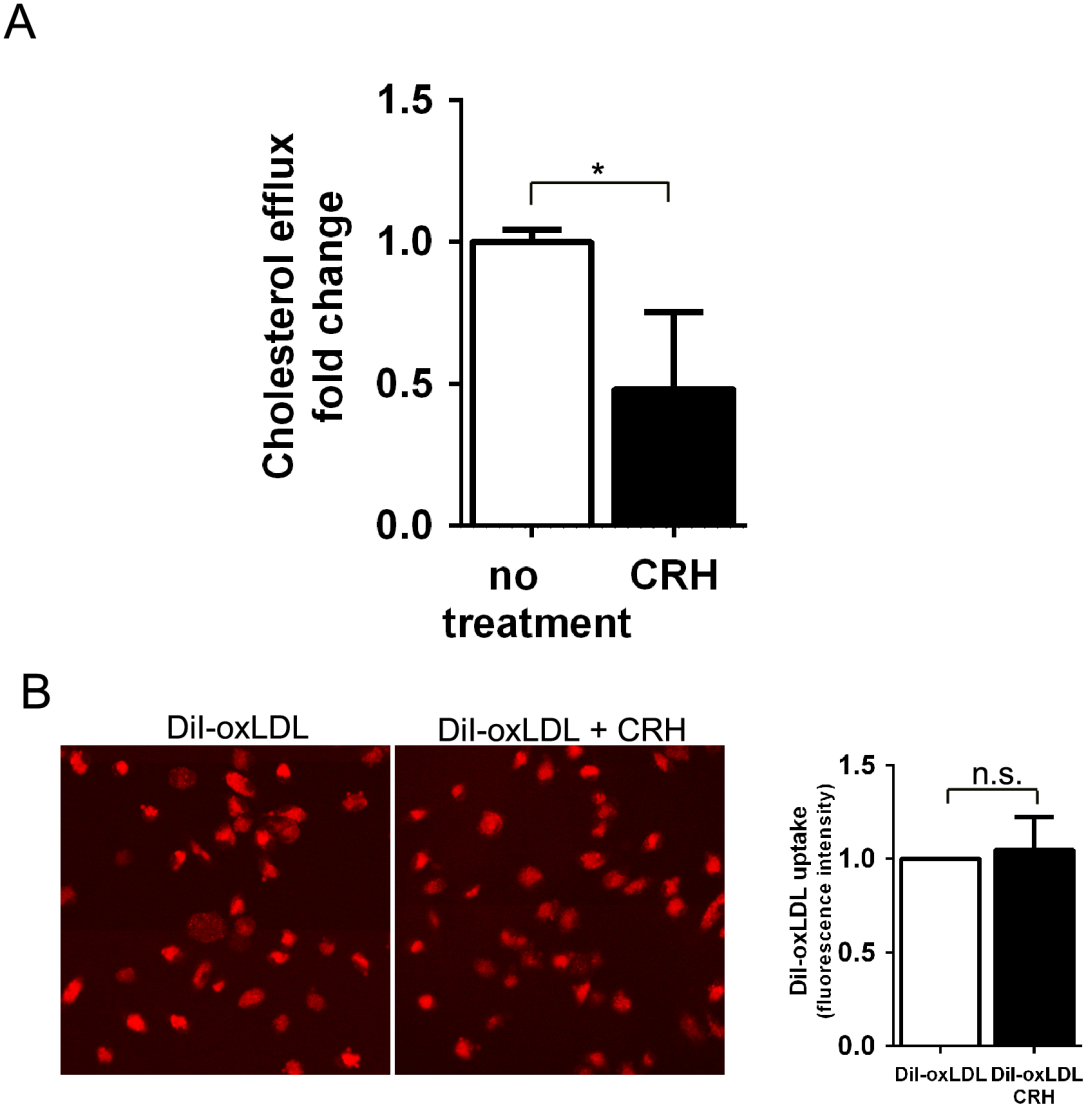
CRH decreases cholesterol efflux. (A) BODIPY-labeled cholesterol efflux assay. Murine peritoneal macrophages treated with or without CRH for 18 hours were labeled with BODIPY-cholesterol, equilibrated with acyl-coA:cholesterol acyltransferase (ACAT) inhibitor-containing medium, and incubated with apoAI. The medium containing BODIPY-cholesterol excreted from the cells was measured with a fluorometer. Results from triplicate experiments were compared. (B) DiI-oxLDL uptake assay. Murine peritoneal macrophages were treated with or without CRH for 18 hours and then exposed to DiI-oxLDL for 30 minutes. Macrophages with DiI-oxLDL uptake were assayed by confocal fluorescence microscopy and flow cytometry to measure the mean fluorescence intensity from the internalized DiI-oxLDL (right graph). Data is presented as mean ± SEM. * P<0.*05*, *n*.*s*.; *no statistical significance*.

Intracellular cholesterol in macrophages is maintained by the balance between uptake and efflux of cholesterol [15]. To assay cholesterol efflux, we loaded BODIPY-conjugated cholesterol onto macrophages treated with or without CRH and used fluorometry to measure the BODIPY-cholesterol exiting the macrophages. The result revealed that CRH treatment induced a 50% decrease in macrophage cholesterol efflux (Fig 4A).

Lipid uptake was measured using a DiI-oxLDL uptake assay. The result showed that DiI-oxLDL uptake was not different between untreated and CRH-treated macrophages (Fig 4B). This suggests that the foam cell-promoting activity of CRH is caused by decreased cholesterol efflux of macrophages.

### CRH down-regulates ABCA1 expression via interaction with CRH receptor 1 (CRHR1) and Akt phosphorylation

**Fig 5:**
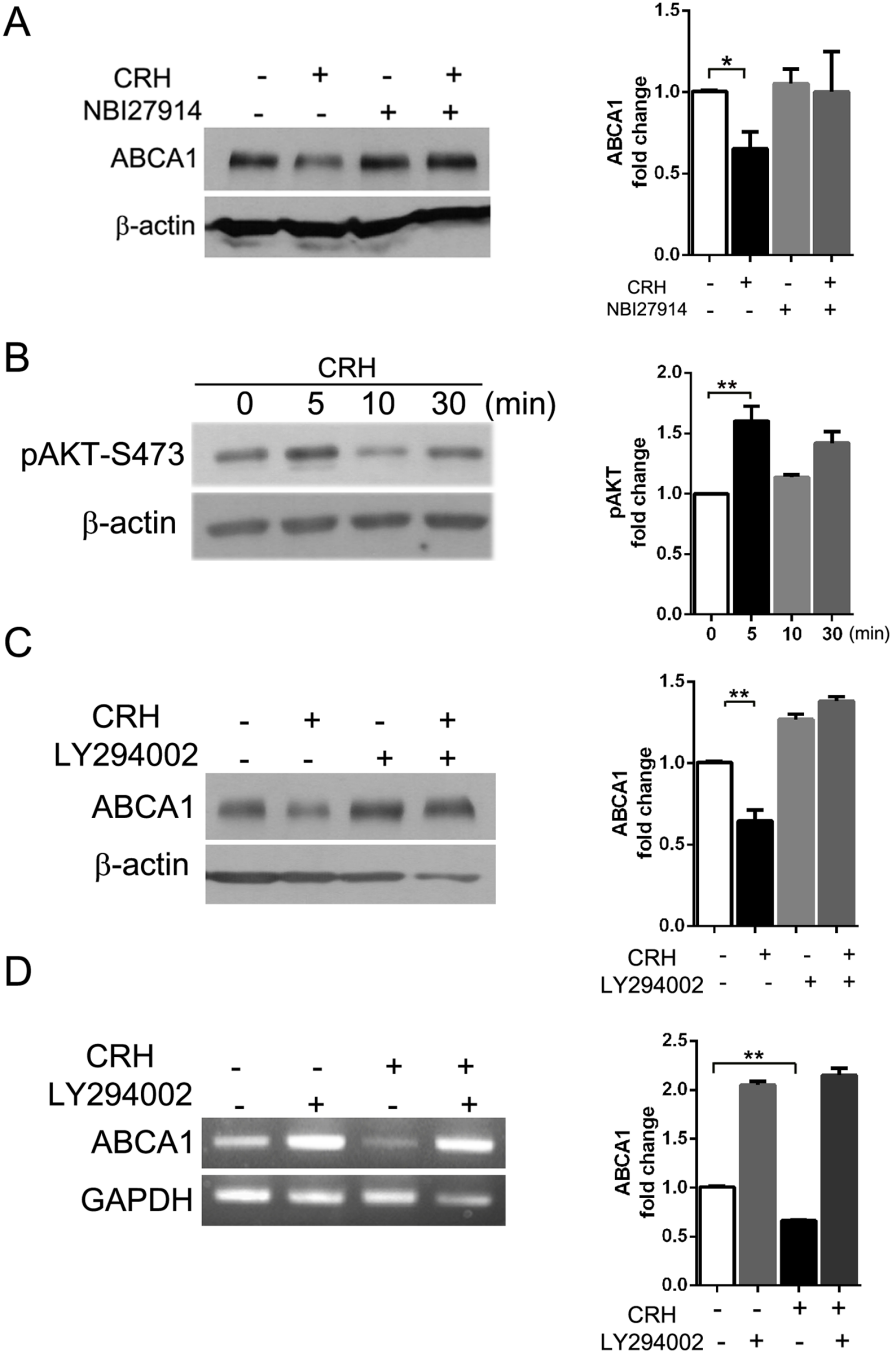
CRH down-regulates ABCA1 expression via interaction with CRH receptor 1 (CRHR1) and Akt phosphorylation. (A) Macrophages were pre-treated with or without NBI27914 (100 nM), a CRHR1-specific blocker. The cells were incubated with or without CRH (10 nM) for 18 hours, lysed and applied to Western blotting for ABCA1. (B) Murine peritoneal macrophages were treated with or without CRH (10 nM) for the indicated times. The cell lysates were applied to Western blotting for phospho-Akt (Ser473). (C) Macrophages pre-treated with or without LY294002 (20 μM) were exposed to CRH (10 nM) for 18 hours and then lysed. Western blotting for ABCA1 was performed with the cell lysates. (D) Semi-quantitative reverse transcriptase PCR for ABCA1 was performed with the macrophages treated as described in (C). All results were derived from triplicate experiments. Quantitative data are presented as mean ± SEM (right graphs, A-D). * P < 0.*05*, ** P < 0.*01*.

We tested whether the down-regulation of ABCA1 was mediated by interaction between CRH and CRH receptor 1 (CRHR1), which is known to be expressed on macrophages [24]. Western blotting for ABCA1 showed that NBI27914, a CRHR1-specific blocker, blocked the effect of CRH on ABCA1 expression (Fig 5A).

A previous report showed that CRH induced phosphorylation of Akt (Ser473) in the human monocyte cell line THP-1 [25]. Our Western blot analysis showed that CRH treatment induced dynamic phosphorylation of Akt (Ser473) in murine peritoneal macrophages (Fig 5B). Phosphorylated Akt (Ser473) was increased 1.6-fold after a 5-minute incubation with CRH (Fig 5B). We determined if phosphorylation of Akt (Ser473) by CRH mediated the down-regulation of ABCA1. We treated macrophages with LY294002, a phosphoinositide 3-kinase (PI3-K)/Akt inhibitor, and exposed the macrophages to CRH for 18 hours. Western blot analysis for ABCA1 showed that LY294002 treatment blocked the effect of CRH on macrophage ABCA1 expression, while untreated cells exhibited decreased expression of ABCA1 in response to CRH (Fig 5C). Furthermore, LY294002-treated macrophages demonstrated increased expression of ABCA1 at baseline (Fig 5C), evidencing the regulating effect of phospho-Akt on ABCA1 expression. Semi-quantitative reverse transcriptase PCR also showed that blockage of Akt phosphorylation by LY294002 prevented the CRH-induced decrease in ABCA1 transcription (Fig 5D).

We concluded that CRH via CRHR1 provokes phosphorylation of Akt (Ser473), and this signaling induces a decrease in LXR-α expression and a subsequent decrease in ABCA1. The CRH-activated pathway promotes macrophage foam cell formation, which is a critical step in atherosclerosis.

## Discussion

Atherosclerosis is characterized by the accumulation of lipids and inflammatory cell infiltrates in the arterial intima and is influenced by various factors [2,26]. A recent study showed that psychological stress and depression are related to increased cardiovascular morbidity and mortality [27–29]. A few studies have sought to elucidate the interconnections between various neurotransmitter systems and cardiovascular function [30–32]. However, the exact mechanism by which psychological stress promotes atherosclerosis remains unclear.

CRH is higher in the blood of patients under psychological stress [6,7]. CRH exists widely throughout brain and peripheral tissues, regulating endocrine, behavioral and immune responses to stress, and thus could be a candidate molecule providing the mechanistic explanation for the psychological etiology of atherosclerosis. CRH targets various immune cell types including mast cells, splenocytes, monocytes/macrophages and lymphocytes, which express CRH receptors [9–13]. CRH augments LPS-induced cytokine production in macrophages and induces expression of toll-like receptor-4 (TLR-4) via activation of transcription factors PU.1 and AP.1 [10,33]. A well-known function of CRH is HPA axis regulation, and hyperactivity of the HPA axis is a characteristic of chronic diseases, including cardiovascular disease [34–36]. Previous studies using intravascular injection or direct administration of CRH into the central nervous system (CNS) revealed the cardiovascular effects of CRH. While intravascular administration of CRH lowered blood pressure through vasodilation [37–39], intracerebroventricular administration of CRH increased heart rate and mean arterial pressure [40]. CRH perfusion in an isolated working heart was shown to increase coronary blood flow and cardiac contractility [41]. A recent study revealed that CRH injection into hypercholesterolemic LDL receptor (LDLR) null mice induced larger atherosclerotic lesion development compared with that in vehicle injection controls [42]. However, the direct mechanism by which CRH promotes atherosclerotic inflammation had not been clarified. Our current study using murine peritoneal macrophages provides this explanation and shows how CRH promotes macrophage foam cell formation, which is the initial and critical step in the development of atherosclerosis.

To study the effect of CRH on macrophages, we used a physiological range of CRH concentration (10–50 nM) [43,44]. Our data indicates that CRH disrupts intracellular cholesterol homeostasis of macrophages and facilitates macrophage foam cell formation (Fig 3). Intracellular cholesterol is maintained through the balance between cholesterol uptake and efflux [15–17]. Our data revealed that CRH-induced foam cell formation is caused by decreased cholesterol efflux, essentially resulting from decreased expression of ABCA1, the key transporter for cholesterol efflux [16]. Efficient cholesterol efflux is pivotal for the prevention of cholesterol accumulation in macrophages; in accordance, our BODIPY-labeled cholesterol efflux assay showed CRH-induced reduction of cholesterol efflux in macrophages (Fig 4A). Our systematic analysis of molecules mediating lipid uptake and efflux using qRT-PCR verified that CRH decreased ABCA1 and SR-B1 in murine macrophages (Fig 1A). ABCA1 is known to mediate the release of excess free cholesterol to apoA1, an extracellular acceptor [16], and SR-B1 is known to mediate alterations in membrane free cholesterol domains and promote free cholesterol efflux independent of extracellular acceptor tethering [45,46] Our ORO staining assay using murine macrophages showed that CRH promoted foam cell formation both in the media with and without apoA1, suggesting the consequences of down-regulations of SR-B1 and ABCA1 (Fig 3, S2 Fig). We do not exclude the possibility that trace amounts of serum cholesterol acceptor resided in the culture plate, affecting the cholesterol efflux of macrophages in the ORO staining experiment without apoA1 (S2 Fig). The foam cell-promoting activity of CRH is not driven by any change in de novo cholesterol synthesis of macrophages, which is evidenced by our experiment using simvastatin-treated macrophages (S3 Fig). We blocked de novo cholesterol synthesis of macrophages and treated the cells with CRH. The experiment showed that CRH augmented intracellular cholesterol in oxLDL-treated macrophages (S3 Fig).

In our experiments, we treated murine macrophages with human CRH and LDL isolated from human plasma based on previous reports, revealing the homology of the molecules between mouse and human [47,48]. Murine CRH peptide is identical to the human CRH peptide at the amino acid level, and the murine CRH gene exhibits greater than 92% homology to human CRH genes within the first 336 nucleotides of the 5' flanking DNA [47,48]. Supporting this, our qRT-PCR using human peripheral blood monocyte-derived macrophages revealed that CRH induced down-regulation of ABCA1 and LXR-α as in murine macrophages (Fig 1). However, in the experiment with human macrophages, SR-B1 level was not changed by CRH (Fig 1B). ABCG1 is a known key cholesterol transporter regulated by LXR- α [15]. In our experiment, ABCG1 level was not significantly decreased by CRH in murine or human macrophages (P>0.05) (Fig 1). Therefore, ABCA1-down-regulation by CRH appears to be corporeal in the foam cell-promoting function of CRH.

Our experiment using a CRHR1 blocker showed that a CRH-induced decrease in ABCA1 depends on CRHR1, which is abundant in macrophage cell membranes [24]. CRH via CRHR1 induced phosphorylation of Akt, and blockade of this pathway abrogated the effect of CRH on ABCA-1 expression (Fig 5).

Our data corresponds to previously reported findings including CRH-induced Akt phosphorylation and the ABCA1 suppressing effect of phospho-Akt [49,50]. A previous study on intestinal inflammation showed that CRH via CRHR1 increased phosphorylation of Akt in human intestinal microvascular endothelial cells [49]. Pregnancy-associated plasma protein-A (PAPP-A), which is found in atherosclerotic plaque, activates PI3-K/Akt signaling and downregulates ABCA1 expression in THP-1-derived macrophage-like cells [50]. Akt phosphorylation by insulin and adiponectin also suppressed ABCA1 expression in human monocyte-derived macrophages [51]. We concluded that CRH-induced phosphorylation of Akt leads to a decrease in ABCA1.

ABCA1 is regulated by LXRs and retinoid-X-receptor (RXR) [52,53]. We showed that CRH induced a decrease in LXR-α RNA and subsequent decrease in ABCA1 in murine and human macrophages (Figs 1 and 2). However, the detailed mechanism of how phosphorylated Akt reduces expression of LXR-α and ABCA1 remains to be elucidated. PAPP-A, as well as CRH, activates PI3-K/Akt signaling and down-regulates LXR and ABCA1, indicating that activation of this pathway suppresses the expression of both LXR and ABCA1 [50].

Macrophage ABCA1 is an important factor that regulates intracellular lipid homeostasis in macrophages and thus prevents atherosclerosis. Previous studies have shown that ABCA1-overexpressing *Apoe* null mice develop less atherosclerosis [54], and *Abca1* null macrophage transplantation into *Ldlr* null mice accelerated foam cell formation and atherosclerosis [55]. As an attempt to increase ABCA1, injection of LXR agonist resulted in the attenuation of plaque formation in *Apoe* null mice [56]. The *in vivo* athero-protective effect of ABCA1 may rely on increased reverse cholesterol transport (RCT); however, changes in cellular cholesterol efflux do not always result in changes in the entire RCT pathway. In previous studies, ABCA1 expression in the endothelial-specific promoter in C57BL/6 mice and ABCA1-overexpressing C57BL/6 mice showed a plasma HDL increase and a reduction of atherosclerosis [57], while ABCA1-overexpressing mice in an Apoe-null background did not show any changes in HDL level [58]. Therefore, analysis to verify if CRH-induced changes in cholesterol efflux affect the entire RCT pathway and atherosclerotic process *in vivo* should be done in future studies. A recent study revealing the anti-inflammatory effect of ABCA1 suggests an athero-protective function of ABCA1 independent of RCT [59] and may suggest an additional explanatory mechanism for athero-promoting effect of CRH.

Our study affirmed the role of ABCA1 in the atherogenic process and verified a mechanism by which CRH as a stress hormone accelerates macrophage foam cell formation, which is a pivotal step in atherosclerosis.

## Supporting Information

S1 Fig CRH does not affect the level of CD36. (A) Murine peritoneal macrophages treated with or without CRH (10 nM) were lysed and applied to the Western blotting for CD36. (B) Quantitative data from the Western blotting are presented as mean ± SEM. *n*.*s*.; *no statistical significance*.
(TIF)

S2 Fig CRH promotes foam cell formation. (A) Murine peritoneal macrophages treated with or without CRH (10 nM) were incubated with or without oxLDL (50 μg/ml) for 18 hours. Oil-red-O (ORO) staining was performed. Representative pictures are provided (Scale Bar; 100 μm). (B) Measurement of ORO absorbance in the extracts of the ORO-stained macrophage. Fold-changes were plotted from triplicate experiments. ** P < 0.01, *** P < 0.001, *n*.*s*.; *no statistical significance*. (C) Intracellular cholesterol measurement. After an 18-hour incubation with or without CRH (10 nM) and oxLDL (50 μg/ml), macrophages were lysed and intracellular cholesterol was measured. *** P < 0.001, *n*.*s*.; *no statistical significance*.
(TIF)

S3 Fig CRH-induced ABCA1 decrease is independent of de novo cholesterol synthesis. (A) Murine peritoneal macrophages treated with or without simvastatin (5 μM), were incubated with or without oxLDL (50 μg/ml), in the presence or absence of CRH (10 nM) for 18 hours. ORO staining and measurement of the ORO absorbance in the macrophage extracts were done as in S2(B). * P < 0.05, ** P < 0.01, *** P < 0.001, *n*.*s*.; *no statistical significance*. (B) Intracellular cholesterol was measured using murine peritoneal macrophages treated as in (A). All results are presented as the mean ± SEM. The results were derived from triplicate experiments. * P < 0.*05*, ** P < 0.*01*, *** P < 0.*001*, *n*.*s*.; *no statistical significance*.
(TIF)
