# Supplementary figures and images for: Corticotropin-Releasing Hormone (CRH) Promotes Macrophage Foam Cell Formation via Reduced Expression of ATP Binding Cassette Transporter-1 (ABCA1)
Source: PLoS One. 2015 Jun 25;10(6):e0130587. doi: 10.1371/journal.pone.0130587 (PMC4481410; doi:10.1371/journal.pone.0130587)

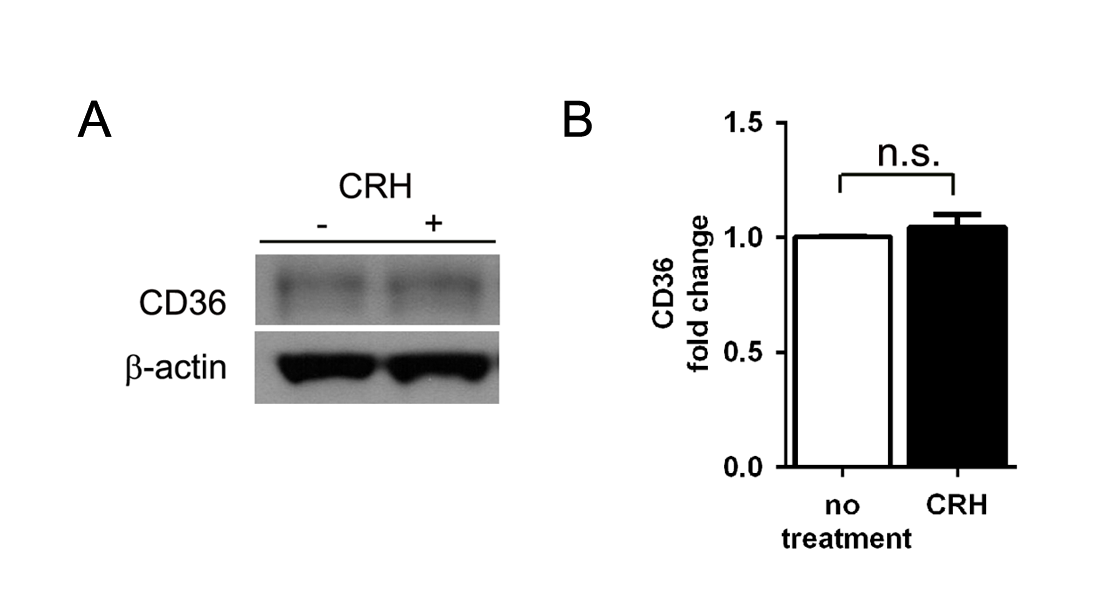

Supplement: S1 Fig — (A) Murine peritoneal macrophages treated with or without CRH (10 nM) were lysed and applied to the Western blotting for CD36. (B) Quantitative data from the Western blotting are presented as mean ± SEM. n.s.; no statistical significance. (TIF) [file pone.0130587.s001.tif]

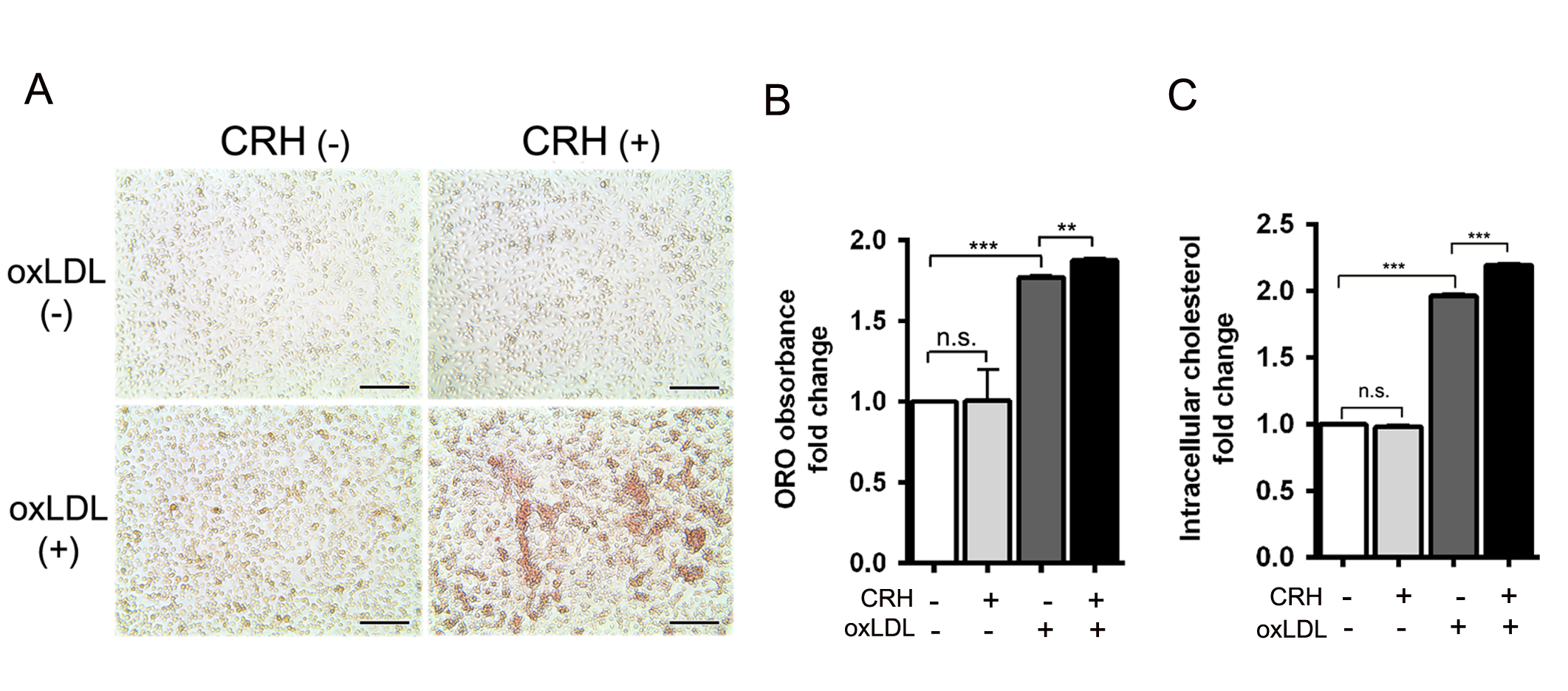

Supplement: S2 Fig — (A) Murine peritoneal macrophages treated with or without CRH (10 nM) were incubated with or without oxLDL (50 μg/ml) for 18 hours. Oil-red-O (ORO) staining was performed. Representative pictures are provided (Scale Bar; 100 μm). (B) Measurement of ORO absorbance in the extracts of the ORO-stained macrophage. Fold-changes were plotted from triplicate experiments. ** P < 0.01, *** P < 0.001, n.s.; no statistical significance. (C) Intracellular cholesterol measurement. After an 18-hour incubation with or without CRH (10 nM) and oxLDL (50 μg/ml), macrophages were lysed and intracellular cholesterol was measured. *** P < 0.001, n.s.; no statistical significance. (TIF) [file pone.0130587.s002.tif]

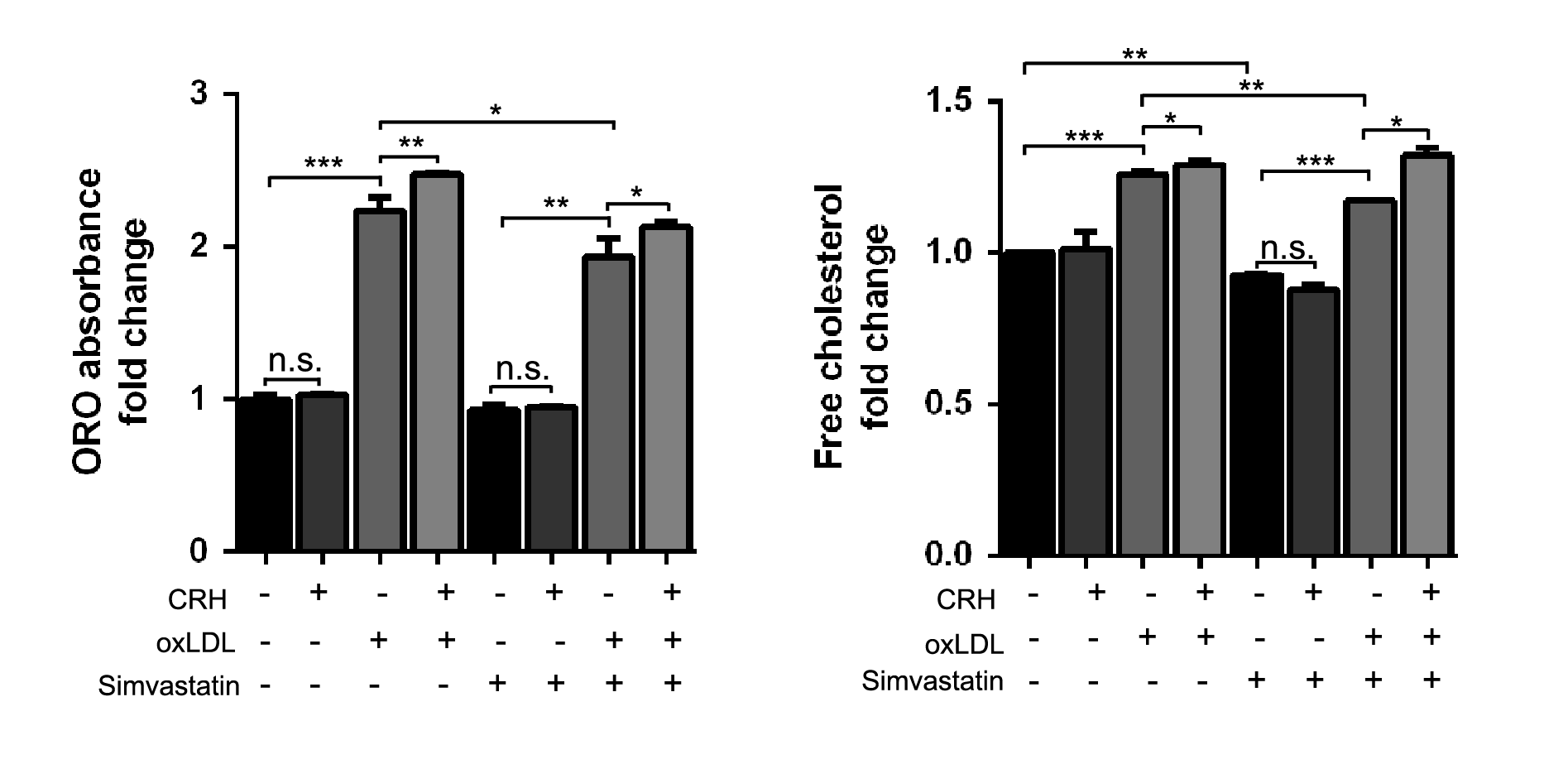

Supplement: S3 Fig — (A) Murine peritoneal macrophages treated with or without simvastatin (5 μM), were incubated with or without oxLDL (50 μg/ml), in the presence or absence of CRH (10 nM) for 18 hours. ORO staining and measurement of the ORO absorbance in the macrophage extracts were done as in S2(B). * P < 0.05, ** P < 0.01, *** P < 0.001, n.s.; no statistical significance. (B) Intracellular cholesterol was measured using murine peritoneal macrophages treated as in (A). All results are presented as the mean ± SEM. The results were derived from triplicate experiments. * P < 0.05, ** P < 0.01, *** P < 0.001, n.s.; no statistical significance. (TIF) [file pone.0130587.s003.tif]
